# A case of infective endocarditis after transurethral prostatic resection

**DOI:** 10.4103/0974-7796.65109

**Published:** 2010

**Authors:** Takashi Kawahara, Hiroki Taguchi, Takuya Yamagishi, Koichi Udagawa, Hideki Ouchi, Hioshi Misaki

**Affiliations:** Department of Urology, Yokohama City University, Graduate School of Medicine, Yokohama, 236-0004, Japan; 1 Department of Urology, Yamato City Hospital, Japan

**Keywords:** Infective endocarditics, transurethral prostatic resection, mitral valve replacement

## Abstract

We report a case of infective endocarditis (IE) after transurethral prostatic resection (TUR-P). A 63-year-old man who had underwent TUR-P for benign prostatic hyperplasia. After 40 days of surgery, he developed a fever. A diagnosis of IE was established by cardiography which detected large vegetation at mitral valve. After intravenous antibiotics therapy, he underwent mitral valve replacement surgery.

## INTRODUCTION

A fever after transurethral endoscopic surgery that involves a transurethral prostatectomy (TURP) is relatively frequently encountered. Therefore, it is common to prophylactically provide an infusion as well as an oral antibiotic after surgery. Normally, in a urinary-tract infection, the fever is reduced at the stage in which there is a quick response to an antibiotic. However, it is often difficult to diagnose or treat Infective endocarditis (IE) after a procedure for the urinary system. We have encountered one case of IE after TURP, and herein report the same.

## CASE REPORT

Patient: 63-year-old male

Major complaint: Fever

Pre-existing history: Mitral valve insufficiency

Family history/lifestyle history: Nothing particularly relevant

### Current medical history

A slight fever had persisted from about June 2008 until 10 days before surgey. In July 2008, the patient underwent a TUR-P for prostatic hyperplasia using 3 days of cefotiam (CTM) i.v. 0.5g ×2/day and was discharged from the hospital on Day 10 after surgery without fever. On about Day 40 after surgery, a fever of 38–39°C was observed, so the patient was suspected of having a urinary-tract infection and was admitted to the hospital.

### Assessment at the time of admission

Body height: 166.3 cm; Body weight: 59.5 kg; Body temperature: 37.8°C; Pulse rate: stable at 84/min; Blood pressure: 116/76 mmHg. Respiratory sound: resonant; Cardiac sound: clear/no noise; Abdomen: flat/soft; Neurological findings: nothing in particular. Laboratory data at the time of admission:

Hematological values: WBC 9000/µL, RBC 4.01 × 106/µL, Hb 10.3 g/dL, Ht 30.5 %, Plt 36.6 × 104/µL , PT 78 %, PT-INR 1.14, APTT 29.4 sec, Fib 628 mg/dL

Biochemistry: TP 6.8 g/dL, Alb 3.2 g/dL, T-bil 0.5 mg/dL, D-bil 0.0 mg/dL, AST 24 IU/L, ALT 35 IU/L, ALP 631 IU/L, γ-GTP 102 IU/L, Ch.E 198 IU/L, LDH 203 IU/L, CK 18 IU/L, Amy 71 IU/L, T-chol 193 mg/dL, TG 80 mg/dL, LDL 122 mg/dL, BUN 9 mg/dL, Cre 0.7 mg/dL, UA 2.4 mg/dL, Na 139 mEq/L, K 4.2mEq/L, Cl 103 mEq/L, Ca 9.4 mg/dL, IP 3.5 mg/dL, Glu 95 mg/dL, HbA1c 5.4%, CRP 5.6 mg/dL

Urinalysis: pH 7.5, RBC many/hpf, WBC many/hpf

### Course after admission

After admission, CTM 0.5 g × 2/day was administered for 3 days, but the CRP did not become negative, so Meropenem (MEPM) 0.5 g × 2/day was administered instead. A tendency for pyretolysis was observed, but because recovery from the urinary-tract infection was slow, echocardiography was performed for the purpose of eliminating endocarditis. In the echocardiogram findings, vegetation was observed, so Ampicillin (ABPC) 2 g × 4/day and Gentamicin (GM) 60 mg × 2/day were administered for four weeks. Pyretolysis and decreased CRP were observed, but the CRP did not become negative. Even after the 4-week administration of antibiotics, the vegetation persisted in the echocardiogram and a possibility of valve failure was suspected, so a valve replacement was performed. Furthermore, the head and chest to pelvic CT did not show any obvious abscess formation.

## DISCUSSION

IE is a systemic disease that normally develops when some type of underlying cardiac disease such as a cardiac valvulopathy exists and presents various clinical symptoms such as symptoms of infection and symptoms of embolization as well as cardiac symptoms. The mechanism of the occurrence of IE is that blood platelets and fibrin attach to a valve that has been damaged by a valvular disease, blood clots adhere thereto, and fibrin blood platelet clots are consequently formed. In addition, it is said that, if transient bacteremia occurs, bacteria attach to the fibrin blood platelet blood clots and form colonies.[1] It is said that the formation of a focus of infection is associated with a high-speed blood flow or a direct collision with the endocardial surface, and onset is rare at a site where no abnormal high-speed blood flow exists.[1]

It is said that the causes of transient bacteremia, which leads to the onset of IE, are mostly associated with surgical procedures in the fields of dentistry, otorhinolaryngology, urology, and obstetrics and gynecology.[2] However, the frequency is low and typical physical findings such as a fever and generalized fatigability are inconsistent, so it is often detected late.[3] Duke's diagnosis criteria are used for the diagnosis of infective endocarditis.[3] Duke's criteria was "two major" or "one major and three minor" or "5 minorcriteria" are needed.

Major criteria is as follows:

1. Persistently positive blood cultures with an organism known to cause endocarditis.
2. Echocardiographically definite vegetation or abscess or new prosthetic dehiscence or new native regurgitation.

Minor criteria is as follows:

1. Predisposing heart condition or IV drug use.
2. Fever > 38°C.
3. Vascular phenomena: arterial emboli, septic pulmonary infarcts, intracranial hemorrhage, mycotic aneurysm, conjunctival haemorrahge.
4. Immunological phenomena: e.g. glomerulonephritis.
5. Positive blood culture, but not meeting major criterion.
6. Raised inflammatory markers.

When making a diagnosis, it is most important to beware of the presence of a heart murmur. Heart murmur is observed in 80 to 85% of all patients with IE of a native valve, and in particular, it is said that the presence of a regurgitant murmur is important.[1] For examinations, simple chest X-rays, echocardiography, and blood culture are important for making a definitive diagnosis. The rate of detection of vegetation is 50% in transthoracic echocardiography (TTE), but it is said that warts can be detected in at least 90% of all patients in transesophageal echocardiography.[4]

In the blood culture, causative bacteria are identified in 70 to 80% of all patients.[5] For causative bacteria, aerobic Gram-positive anaerobic bacteria account for 70 to 80%, and it is said that *Staphylococcus aureus, Streptococcus viridans, enterococcus, Coagulase-negative staphylococcus*, etc., are frequently observed.[6,7] In the present case, a blood culture was performed twice, and enterococcus was detected both times.

As for treatment, according to the guidelines from the American Heart Association (AHA), sensitive antibiotics should be administered for 4 to 6 weeks. However, if there is no response to the administration of antibiotics, the heart failure symptoms are severe, infection has spread around the valve, or there is a large wart measuring least 1 cm in size, then a valve replacement is performed.[8]

In the present case, because of the pre-existing history of mitral valve insufficiency, it is believed that a high-speed blood flow was present in the endocardial surface before the surgery. The rate of occurrence of central nervous system complications during the course of IE is 20 to 45%, and cerebral embolization is most frequently observed.[9] The sequelae when a central nervous system complication appears are often serious, and it is believed that it would be necessary to proactively perform an examination, such as a echocardiography as a measure against postoperative fever.
